# On Some Aspects of Memory

**Published:** 1901-01-26

**Authors:** John Aikman


### ON SOME ASPECTS OF MEMORY.

By JOHN AIKMAN, M.D.

### II.—GEOGRAPHY AND TIDAL INFLUENCE.

(*Continued.*)

The pressure to which the surface of his body is subjected is therefore about 15½ tons. When some cause concentrates that pressure upon a particular cell or group of cells in his brain we recognise the printing force of an impression made upon his memory.

The same reflection explains the depressing or exhilarating effect of changes in the barometer upon our daily burden.

But while the blood vessels of the brain are saved the pumping power of the heart, they are not free from conditions of dilatation and contraction.

Dr. Lange,[3] of Copenhagen, has pointed out that our emotions are chiefly associated with these conditions of the blood vessels in our brain.

The emotions which tend towards joy are those in which the blood vessels are full and dilated. In suspense the vessels are contracted. In anger or sorrow there is alternate contraction and dilatation, followed by a sense of weariness. In despair and disappointment there is neither contraction nor dilatation, but merely a want of tone and no permanent record. Rhythm is restful. A breach of rhythm is the factor of surprise, or its playfellow comicality. Sympathy is, like the chording of music, a doubled sensation, incomplete in the individual; attentive and hopeful.

We come very near the limit of our knowledge when we recognise these facts. We have travelled up to them quickly and there is some detail to fill in.

What circumstances affect the nutrition of the brain? Its blood supply must be plentiful, and of good quality. If it is not plentiful a man faints, and the making of memory is lost. He loses the power to stand upright and falls to the ground. This unconscious, unwilling act sends the blood back to the brain on the same principle as water runs down a hill; and consciousness returns. The commonwealth has secured its existence by concession.

During sleep the brain has a lessened supply of blood, and memory is in abeyance. It returns, without the domination of reason, during dreams, when some half-hidden cause rouses the blood supply. The blood flush, not directed by special or common sense, wanders among the more permanent or more recent pathways and brings into disorderly form "the stuff that dreams are made of."

Fear brings a dream of a different sort. The face is pale, and the blood vessels of the brain are without tone. The brain cells are ready to take impressions, but the special senses are startled rather than alert, and there is no deep record. There is blood enough, nutriment enough, but neither direction nor concentration. The very vagueness of fear prevents a permanent impression.

Therein lies the difference between fear and courage. In the presence of danger, when met in coolness, an impression may be made which lasts a lifetime. There is concentration for action, and memory is the record of activity. The trembling muscle, the unsteady knee, the palpitating heart, and the choking throat of fear, all tell the story of unpreparedness. The tense muscle, the pale determined face, the clear eye, and the steady heart-beat of courage indicate preparedness, and only relax, and perhaps run wild, when the danger is past.

That gross impersonation of fear, the horrors of drink, is an example of how the brain cells are affected by an altered *quality* of the biood. There is no equal terror in life. The timid mouse, itself unreal, rules the man and arouses consternation in an intellect of at least average standard. The common drunkard does not so often suffer in this way. He has no vivid memory to rouse. The man who does think, and who does outrage to his thinking power by the abuse of alcohol, pays the penalty of a present hell. The objects of his fear are unreal. He has lost even the power of recognising his surroundings. His uptake, or faculty of observation, is gone. On the other hand his memory is active, even if disordered.

Mr. Victor Horsley[4] states this question more strongly in the light of recent experiment.

He shows that, even in dietetic doses, the stimulation of alcohol is unreal. That it almost immediataly shows what is called the "reaction time"—that is to say the time which intervenes between observation and action. He made experiments with compositors setting up type, and he found that they worked quicker and with greater accuracy when no alcohol had been taken.

He admits the apparent increase of activity, but claims it as an increase in the same kind as the "racing of an engine when the governor is removed."

We must, however, be careful not to push the charge against alcohol too far. Poets in prose and verse, novelists, historians, and playwriters, have used, and even abused, alcohol. Shakspere is said to have died of drink. Burns we know is not free from the imputation. To one class of mind stupidity and sleep is a Nirvana. Alcohol supplies both. To another class of mind "it rounds the corners of life," as Matthew Arnold puts it, and makes poetry out of a common humanity. To attain this result the mind must have unclouded periods for observation till it makes up its brain capital, and wait long for a warrant to declare a dividend. "In vino veritas" may be true, but only when we have some truth in us.

Opium and the other narcotic drugs fall under the same category. But more than the perfect English of a De Quincey, or the poetry of a Coleridge should be needful to tempt us to "race like an engine when the governor is removed."

From ages and stages which are dubiously criminal follow me to one of perfect innocency. You have heard a baby cry. A young baby does not shed tears. Darwin, in his marvellous book on the "Expression of the Emotions in Man and in Animals," fixes the date of the first tear between the second and the fourth month. Some education of the brain is needed to rouse the tear gland to activity. Now, the eye is simply an exposed portion of the brain. It gets its blood from the blood vessels which directly feed the brain. The same source supplies the tear-producing gland.

It is not only poetry but fact that the flow of tears lessens the tension of the blood in the brain. You remember that when "Home they brought her warrior dead," "She must weep or she will die."

[3] Brit. Med. Jour., Feb. 17, 1900, p. 403. [4] Lee and Raper Lecture, St. James's Hall, April 27, 1900; Lancet May 5, 1900.

